# Multiple enzyme studies in the vaginal fluid in relation to benign and malignant gynaecological conditions.

**DOI:** 10.1038/bjc.1966.55

**Published:** 1966-09

**Authors:** G. G. Muir


### MULTIPLE ENZYME STUDIES IN THE VAGINAL FLUID IN RELATION TO BENIGN AND MALIGNANT GYNAECOLOGICAL CONDITIONS

G. G. MUIR

*From the Department of Chemical Pathology, St. Bartholomew's Hospital, London, E.C.* 1.†

Received for publication April 28, 1966.

ODELL and Burt's work (1949) on the beta glucuronidase content of vaginal fluid has stimulated considerable interest in the use of enzymes as definitive tests for the diagnosis of carcinoma of the cervix. Bonham and Gibbs (1962) published their results on 6-phosphogluconate dehydrogenase content of the vaginal fluid. In the course of a series to evaluate the use of this enzyme other enzymes were studied.

Since this work was part of an evaluation of 6-phosphogluconate dehydrogenase, the study of these enzymes has confined itself solely to their application under the conditions used for 6-phosphogluconate dehydrogenase. It must be remembered that such conditions may not be suitable for these enzymes and result in a poor recovery. Since one is studying the effects of freezing drying in releasing intracellular enzymes, the effect of freeze drying on a pure enzyme is not a satisfactory estimate of the recoveries.

It was felt that the situation in the posterior fornix was comparable to that occurring with effusions, where a malignant epithelium is in contact with an exudate or secretion. In general the enzymes chosen for study were those which had been shown to be raised in malignant effusions, or else enzymes which had been shown to be raised in neoplastic materials, or enzymes which had been found to be raised in the serum from cases with neoplasia.

*Lactic dehydrogenase*

Wroblewski and Wroblewski (1958) and Fleisher (1959) showed that the lactic dehydrogenase activity of malignant pleural or ascitic fluid was raised above serum activities. Horrocks, King, Waind and Ward (1962) confirmed this finding, but also pointed out that the enzyme was raised in inflammatory effusions. Barrett and Gibson (1964) have shown that the isoenzyme 5 of lactic dehydrogenase is raised in breast tissue and the higher the tumour grading the more isoenzyme could be detected. Latner (1964) confirmed similar findings in cases of carcinoma of the cervix.

In patients with carcinomatosis, lactic dehydrogenase is increased in between 40% and 50% (Hill and Levi, 1954 ; Bierman *et al.*, 1957).

*Aldolase*

Sibley and Lehninger (1949) and White (1958) found that the serum aldolase was increased in 20% of cases with carcinomatosis. Warburg and Christian

* This work is part of an M.D. Thesis submitted to the University of London

† Present address : Department of Pathology, Bedford General Hospital (South Wing), Kempston Road, Bedford.

(1943) found that in rats with Jensen sarcoma the serum aldolase was increased; the increase only occurred if the tumour were large, then it was proportional to size. Sibley and Lehninger (1949) showed that the serum aldolase fell after removal of the neoplasm.

*Glutamic oxaloacetic transaminase*

Unless there are secondary deposits in the liver, the serum oxaloacetic transaminase is rarely found to be elevated in patients with carcinomatosis. In work on animals with the Morris hepatoma 5231, Dyer *et al.* (1961) showed that the glutamic oxaloacetic transaminase tended to be raised. This elevation fell when the tumour was removed. In a more rapidly growing hepatoma, the tumour content of glutamic oxaloacetic transaminase was lower than in the slowly growing Morris hepatoma.

### MATERIALS

Samples were obtained from women attending the Well Women's Clinic at St. Bartholomew's Hospital and the Gynaecological Departments of that hospital, the Luton and Dunstable Hospital and Bedford General Hospital. In this paper the women were only classified as normal if there were no detectable cytological or gynaecological lesion.

In the discussion of the enzyme results the following terms are used:

1. Detectable activity. This is used to mean an activity being present, but not to such a level as to suggest malignancy.
2. Elevated activity; this means an activity above the diagnostic level.

### METHOD

Samples were obtained in a manner similar to that of Bonham and Gibbs (1962); 6-phosphogluconate dehydrogenase was assayed by the methods of Glock and McLean (1953).

*Lactic dehydrogenase*

The assay of the lactic dehydrogenase content was a modification of the method of Wroblewski and LaDue (1955). The reagents were all obtained in a kit from the Boehringer Corporation. The assay was scaled down to be suitable for vaginal fluid.

Calculation:

The results were expressed for convenience in Wroblewski units.

$$\therefore \frac{x}{0{\cdot}001} \times \frac{1000}{y} = \text{units per gram dry weight}$$

where x = the change in optical density per minute, y = the mg. dry weight of vaginal fluid in the reaction mixture.

*Glutamic oxaloacetic transaminase*

The method used was a modification of the ultraviolet assay described by Karmen, Wroblewski and LaDue (1955). The reagents were all obtained in a kit from the Boehringer Corporation.

Calculation :

Wroblewski units were used. If x = the recorded change per minute, and y = the mg. of vaginal debris present in the reaction mixture, the activity per gram is given by the following formula :—

$$\text{units per gram} = \frac{x}{0{\cdot}001} \times \frac{1000}{y}$$

*Aldolase*

A modification of the ultraviolet method of Bruns (1954) was used. In this assay the enzyme activity is determined by linking the reaction to the ultraviolet by the use of triose phosphate isomerase and glycerophosphate dehydrogenase. Bruns unit is defined as that amount of enzyme activity which removed the 0·0446 $\mu$moles of fructose1–6 diphosphate per hour at 25° C. In this assay, 0·892 $\mu$moles of nicotinadenine dinucleotide are reduced during the conversion of 0.0446 $\mu$moles of fructose 1–6 diphosphate to triose phosphate.

The overall reaction is fructose1–6 diphosphate + 2 nicotinamide adenine dinucleotide (reduced)
→2 glycerol—1-phosphate + 2 nicotinamide adenine dinucleotide.

In a 3 ml assay the optical extinction of 0·0892 $\mu$moles nicotinamide adenine dinucleotide phosphate reduced form is 0.18732 optical density units. Thus a change of optical density of 0·18732 optical density units in 1 hour equals 1 Bruns unit.

If x = the change in 20 minutes, and y = the mg. of vaginal debris in the assay mixture, the Bruns unit per gram will be given by the following formula :

$$\text{Bruns unit per gram} = \frac{x}{0{\cdot}187} \times 3 \times \frac{1000}{y}$$

*Glucose-6-phosphate dehydrogenase*

Glock and McLean (1953) described two methods for the assay of glucose-6-phosphate dehydrogenase. One of the methods has the advantage that it allows the comparison of the two enzymes at pH 7·6. The assay was used. Reagents and materials are modified to suit this assay in the vaginal fluid.

0·5 ml. 0·1 molar magnesium chloride
0·5 ml. 0.25 molar glycylglycine buffer, pH 7·6
0·2 ml. of a solution containing
    0·2 molar trisodium 6 phosphogluconate
    0·2 molar glucose-6-phosphate sodium salt
0·1 ml. vaginal fluid supernatent

The reaction is started by the addition of 0·2 ml. nicotinamide adenine dinucleotide phosphate 10 mg./ml. The blank has 0.2 ml. of distilled water instead of the co-enzyme.

The second cell consists of the same reaction mixture, but contains only 6-phosphogluconate. The difference between the activity from cell one and two gives a measure of the activity due to glucose-6-phosphate dehydrogenase.

### RESULTS

### (a) *Lactic dehydrogenase*

Fig. 1 shows the results in 25 normal women compared with 6-phosphogluconate dehydrogenase in the same specimen. 80% (20) of the vaginal samples gave lactic dehydrogenase activities below 30,000 units per gram dry weight. 16% of samples (4 cases) gave elevated levels of 6-phosphogluconate dehydrogenase, in a previous paper describing a larger series, 5·8% of normal women had elevated levels (Muir and Canti, 1966).

TABLE I.—*Vaginal fluid activities of lactic dehydrogenase and 6-phosphogluconate dehydrogenase in menstruation*

| Day | 6-phosphogluconate dehydrogenase units per gram | Lactic dehydrogenase units per gram |
|---|---|---|
| 2 | 101 | 20,000 |
| 4 | 81·5 | 46,000 |
| 2 | 105 | 70,000 |
| 5 | 199 | 68,000 |
| ? | 102 | 30,000 |
| 3 | 323 | 65,000 |

Table I gives the figure in menstruating women. Both enzymes are elevated in menstruation, but the lactic dehydrogenase does not appear so markedly elevated. Two of 6 cases had a lactic dehydrogenase level of 30,000 units per gram dry weight or less.

TABLE II.—*A comparison of lactic dehydrogenase and 6-phosphogluconate dehydrogenase in benign gynaecological conditions*

| Enzyme | Number of cases | No detectable activity | Detectable activity | Percentage | Over level | Percentage |
|---|---|---|---|---|---|---|
| 6-phosphogluconate dehydrogenase | 43 | 13 | 30 | 69·8 | 21 | 48.8 |
| Lactic dehydrogenase | 43 | 6 | 37 | 86 | 20 | 46·5 |

In benign gynaecological lesions (Table II) both enzymes give similar incidence of elevated enzyme results. From Fig. 1 it is apparant that lactic dehydrogenase is more often present in normal vaginal fluid than 6-phosphogluconate dehydrogenase. This difference between the two enzymes would explain the higher incidence of detectable lactic dehydrogenase in cases with gynaecological lesions.

In cases of trichomonas vaginalis both enzymes were affected to a similar degree. There was no sample without elevated enzyme levels. From Table II it is apparent that while it is possible to have an elevated lactic dehydrogenase without an elevated 6-phosphogluconate dehydrogenase, the other distribution is more common.

Table III gives an analysis according to the different types of carcinoma. In carcinoma *in situ*, invasive cervical carcinoma, and carcinoma of the body, the two enzymes give similar results. 6-phosphogluconate dehydrogenase activity gives a better correlation with carcinoma of the ovary than lactic dehydrogenase If the two enzymes had been together, 3 of 4 carcinomas *in situ* would have been

FIG. 1.—Lactic dehydrogenase levels in normal women.

TABLE III.—*A comparison of the vaginal fluid activities of lactic dehydrogenase and 6-phosphogluconate dehydrogenase in varying gynaecological malignancies*

| Malignancy | Number | Enzyme | No activity detected | Activity detected | Diagnostic activity |
|---|---|---|---|---|---|
| Carcinoma | 4 | 6PGD | 1 | 3 | 2 |
| *in situ* | | LDH | 0 | 4 | 2 |
| Carcinoma of | 14 | 6PGD | 0 | 14 | 14 |
| the cervix | | LDH | 0 | 14 | 13 |
| Carcinoma of | 6 | 6PGD | 0 | 6 | 4 |
| corpus uteri | | LDH | 0 | 6 | 4 |
| Carcinoma | 4 | 6PGD | 1 | 3 | 2 |
| of ovary | | LDH | 0 | 4 | 1 |

detected, and 5 of 6 body carcinomas. No difference would have occurred with ovarian carcinomas.

### (*b*) *Glutamic oxaloacetic transaminase*

Vaginal fluid oxaloacetic transaminase was studied in 63 women with no evidence of malignancy and in 20 women with malignant disease of the genital tract.

Fig. 2 shows the results in normal women. Twenty-one normal women were studied. Two of 21 women had 6-phosphogluconate dehydrogenase above 80 units per gram dry weight. Since only 2 women (5%) had glutamic oxaloacetic transaminase of more than 2000 units per gram dry weight, it was decided that 2000 u/g. dry weight should be the upper limit for the normal range.

Fig. 3 shows the results in 42 women with gynaecological lesions. Nineteen had glutamic oxaloacetic transaminase of 2000 units per gram dry weight or below, while 21 women had results of less than 80 units per gram dry weight with 6-phosphogluconate dehydrogenase.

FIG. 2.—Oxaloacetic transaminase in normal women

TABLE IV.—*A Comparison of 6-Phosphogluconate Dehydrogenase and Glutamic Oxaloacetic Transaminase in the Vaginal Fluid of Gynaecological malignancy*

| Malignancy | Number | Enzyme | No activity detected | Activity detected | Diagnostic activity |
|---|---|---|---|---|---|
| Carcinoma | 1 | 6PGD | 1 | — | — |
| *in situ* | | GOT | — | 1 | — |
| Carcinoma | 14 | 6PGD | — | 14 | 11 |
| of cervix | | GOT | 1 | 13 | 11 |
| Carcinoma | 3 | 6PGD | — | 3 | 1 |
| of body | | GOT | — | 3 | 2 |
| Carcinoma | 2 | 6PGD | 1 | 1 | 1 |
| of ovary | | GOT | 1 | 1 | 1 |

Table IV shows the results in 20 cases of gynaecological carcinoma. While 5 cases were not detected with 6-phosphogluconate dehydrogenase, 7 were not detected with glutamic oxaloacetic transaminase. In this small series the two enzymes gave similar results, though 6-phosphogluconate dehydrogenase correlated better with malignancy

FIG. 3.—Oxaloacetic transaminase levels in women with gynaecological lesions.

(*c*) *Aldolase*

There were 21 normal women in this group. Fig. 4 shows the results. Of the 21 women, only one woman had a level of more than 2,500 units, thus 95% of these women fell under 2,500 units per gram dry weight. It was decided that this level should be regarded as the upper limit of normal. Table V shows the results in menstruation. In menstruation the vaginal fluid aldolase activity is not as markedly increased as the 6-phosphogluconate dehydrogenase activity.

TABLE V.—*Vaginal Fluid Activities of Aldolase and 6-Phosphogluconate Dehydrogenase During Menstruation*

| Day | 6-phosphogluconate dehydrogenase units per gram | Aldolase units per gram |
|---|---|---|
| 4 | 81·5 | 194 |
| 2 | 105 | 705 |
| 3 | 408 | 960 |
| 4 | 551 | 1,730 |
| 5 | 599 | 4,160 |

Fig. 5 shows the results in a group of women with gynaecological lesions. Of 43 women, 25 have 6-phosphogluconate dehydrogenase activities greater than 80 units per gram dry weight, while 20 had vaginal aldolase contents of more than 2,500 units per gram dry weight.

Fig. 4.—Aldolase in normal women.

Fig. 5.—Aldolase in women with gynaecological lesions.

In 3 cases with trichomonas vaginalis infection, the vaginal aldolase activity was increased in only one, while the vaginal 6-phosphogluconate dehydrogenase was increased in all cases. It appears that the vaginal fluid aldolase contents gives a slightly lower incidence of false positive results than 6-phosphogluconate dehydrogenase.

The results in cases of malignancy are given in Table VI. Of 4 samples from cases of carcinoma *in situ*, both enzymes failed to detect two cases. In established invasive carcinoma of the cervix, uterus, and ovary, the 6-phosphogluconate dehydrogenase activity correlated more closely with malignancy.

TABLE VI.—*A Comparison of 6-phosphogluconate dehydrogenase and Aldolase in the Vaginal Fluid of Gynaecological Malignancy*

| Malignancy | Number | Enzyme | No activity detected | Activity detected | Diagnostic activity |
|---|---|---|---|---|---|
| Carcinoma | 4 | 6PGD | 1 | 3 | 2 |
| *in situ* | | Aldolase | — | 4 | 2 |
| Carcinoma of cervix | 12 | 6PGD | — | 12 | 10 |
| and vagina | | Aldolase | — | 12 | 8 |
| Carcinoma | 6 | 6PGD | — | 6 | 5 |
| of corpus | | Aldolase | — | 6 | 4 |
| Carcinoma | 4 | 6PGD | 1 | 3 | 3 |
| of ovary | | Aldolase | 1 | 3 | 1 |

(*d*) *Glucose-6-phosphate dehydrogenase*

The results on 23 samples are given in Table VII. There were 12 samples from malignant or pre-malignancy conditions. Among these 12 cases there were 8 established carcinomas, in 6 of which glucose-6-phosphate dehydrogenase was elevated to an equal or greater extent than 6-phosphogluconate dehydrogenase at pH 7·6 and pH 9·0.

In 11 samples from patients with benign conditions are shown only 3 cases in which glucose-6-phosphate dehydrogenase was higher than 6-phosphogluconate dehydrogenase.

There is no evidence that the use of glucose-6-phosphate dehydrogenase increases diagnostic efficiency. One sample from an established carcinoma was missed by 6-phosphogluconate dehydrogenase, while 3 established carcinomas were missed by glucose-6-phosphate dehydrogenase using a similar level of activity to distinguish benign and malignant conditions.

(*e*) *Simultaneous multiple enzyme studies*

In a certain number of samples more than 2 enzymes were studied.

*Normal women.*—In 6 samples from normal women, glutamic oxaloacetic transaminase and 6-phosphogluconate dehydrogenase, lactic dehydrogenase, and aldolase activities were estimated. In a further 10 cases the oxaloacetic transaminase, the 6-phosphogluconate dehydrogenase and the aldolase were studied. In 3 samples the activities of 6-phosphogluconate, lactic dehydrogenase and aldolase were also studied. In only 1 sample was the 6-phosphogluconate dehydrogenase and the other enzymes elevated . In 2 of these samples, the glutamic oxaloacetic transaminase was elevated by itself.

TABLE VII.—*A Comparison of Glucose-6-phosphate Dehydrogenase and 6-Phosphogluconate Dehydrogenase Activities in Benign and Malignant Conditions*

| Clinical Condition | Enzyme activity in units per gram dry weight: glucose-6-phosphate dehydrogenase pH 7·6 | 6-phosphogluconate dehydrogenase pH 7·6 | 6-phosphogluconate dehydrogenase pH 9·0 |
|---|---|---|---|
| Carcinoma of the cervix | 62·2 | 26·3 | 16·7 |
| a) before irradiation | 555 | 421 | 167 |
| b) post irradiation | | | |
| Carcinoma of cervix IV | 3020 | 1770 | 2300 |
| Carcinoma of cervix | 373 | 278 | 388 |
| Carcinoma of cervix | 76 | 498 | 709 |
| Carcinoma of vagina | 44·1 | 99·4 | 114 |
| Pelvic carcinomatosis | 468 | 343 | 402 |
| Carcinoma of body | 870 | 522 | 504 |
| Chorion epithelioma | 123 | 187 | 119 |
| Dysplasia | 789 | 1095 | 861 |
| Dysplasia | 101 | 263 | 430 |
| Trichomonas vaginalis | 105 | 153 | 97·1 |
| Fibroids and cervical polyp | 4·8 | nil | 38·2 |
| Postmenopausal bleeding | 263 | 115 | 86·1 |
| Low oestrogen level | 38 | 206 | 201 |
| Low oestrogen level | 24·0 | 52·6 | 49 |
| Chronic cervicitis | 1446 | 655 | 742 |
| Erosion | 359 | 230 | 259 |
| Postmenopausal bleeding | 114 | 173 | 205 |
| Post abortion | 53 | 273 | 340 |
| Post cone biopsy | 33 | 192 | 177 |
| Chronic cervicitis | 207 | 512 | 505 |

*Women with gynaecological lesions.*—In 17 samples the 6-phosphogluconate dehydrogenase, the aldolase, glutamic oxaloacetic transaminase and lactic dehydrogenase were all studied. In 11 samples, 6 phosphogluconate, glutamic oxaloacetic transaminase and aldolase were studied. In the remaining six cases 6 phosphogluconate dehydrogenase, lactic dehydrogenase and glutamic oxaloacetic transaminase activities were also studied. In only 1 case were any other enzymes elevated when the 6-phosphogluconate dehydrogenase was not. In 18 of 34 samples the 6-phosphogluconate dehydrogenase was elevated. Similar results were found with the glutamic oxaloacetic transaminase. In 11 of 23 samples the lactic dehydrogenase was elevated while in 12 of 28 cases the aldolase was elevated. Each enzyme gave a false positive rate of 48% for lactic dehydrogenase, 43% for aldolase, 53% for glutamic oxaloacetic transaminase and 6-phosphogluconate dehydrogenase. It was also found that if samples had an elevated 6-phosphogluconate dehydrogenase, in 58% of these samples the lactic dehydrogenase was also elevated, while in 55% of the same sample the glutamic oxaloacetic transaminase was elevated. In 53% the aldolase was elevated. If the lactic dehydrogenase activity in the samples was raised, in 54% of these samples the 6-phosphogluconate dehydrogenase was elevated, in 53% the glutamic oxaloacetic transaminase, and in 54% the aldolase. If the glutamic oxaloacetic transaminase was elevated the 6-phosphogluconate dehydrogenase was elevated in 55%; the lactic dehydrogenase in 54% and the aldolase in 57%. When the aldolase was elevated the 6-phosphogluconate dehydrogenase was also elevated in 53%; the glutamic oxaloacetic transaminase in 57% and the lactic dehydrogenase in 55%. Table VIII summarises these results. From Table VIII it appears that in benign

TABLE VIII.—*Correlation of Multiple Enzyme Studies in Benign Conditions*

| Enzyme elevated | Number of sample | Associated enzyme elevated with number of samples which might have shown elevation: Lactic dehydrogenase | Aldolase | Oxaloacetic transaminase | 6-phospho-gluconate dehydrogenase |
|---|---|---|---|---|---|
| 6-phospho-gluconate dehydrogenase | 18 | 7 of 12 58% | 8 of 15 53% | 10 of 18 55% | — |
| Lactic dehydrogenase | 11 | — | 5 od 9 45% | 7 of 11 63% | 6 of 11 54% |
| Oxaloacetic transaminase | 18 | 9 of 14 64% | 8 of 14 57% | — — | 10 of 18 55% |
| Aldolase | 12 | 5 of 9 55% | — | 8 of 14 57% | 8 of 12 67% |

conditions the lactic dehydrogenase and the glutamic oxaloacetic transaminase activities seemed to correlate together while the 6-phosphogluconate dehydrogenase and the aldolase appear also to correlate with each other.

TABLE IX.—*Mixed Enzymes in the Vaginal Fluid of Women with Carcinoma of the Cervix*

| Enzyme units per gram dry weight: 6-phosphogluconate dehydrogenase | Serum glutamic oxaloacetic transaminase | Lactic dehydrogenase | Aldolase |
|---|---|---|---|
| 1920 | 10,000 | 70,000 | 22,720 |
| 2020 | 3,000 | 330,000 | 13,120 |
| 565 | 2,200 | 60,000 | 5,410 |
| 1830 | 2,800 | 260,000 | 7,540 |
| 1280 | 5,400 | 100,000 | 1,920 |
| 2040 | 7,000 | 95,000 | 6,276 |
| 537 | 6,200 | 39,000 | 9,410 |
| 225 | 3,000 | 80,000 | 6,009 |
| 394 | 800 | 42,000 | 18,200 |
| 28·8 | 400 | 3,000 | 1,185 |
| 2480 | 2,400 | 150,000 | 11,610 |
| 1560 | — | 700,000 | 1,845 |

*Women with gynaecological malignancies*.—In 11 samples of invasive carcinoma of the cervix, the 4 enzymes were studied together, and in one case the oxaloacetic transaminase was not assayed. The results are shown in Table IX. In one sample the 6-phosphogluconate dehydrogenase was not elevated, but neither were any of the other enzymes. In Table X the results are summarised.

In Table XI the results of multiple enzymes in carcinoma of the corpus uteri and ovary are given. In 3 samples from carcinoma uteri 4 enzymes were studied and in 2 samples, 3 enzymes. In only one case was the aldolase diagnostic when the 6-phosphogluconate dehydrogenase was not. In one case oxaloacetic transaminase was the only elevated enzyme. If the 6-phosphogluconate dehydrogenase was elevated, the other enzymes were generally elevated.

Had all 4 enzymes been used, the 5 cases would have been detected. When only the 6-phosphogluconate dehydrogenase, lactic dehydrogenase, and aldolase were used, 4 cases were diagnosed. Individually, of these 3 enzymes, each did as well as the other.

TABLE X.—*Results of Multiple Enzymes in Invasive Carcinoma of the Cervix*

Note : The table compares the number of elevations of one enzyme with the number of possible elevations it could have in association with another enzyme.

| | | Associated enzymes elevation | | | |
|---|---|---|---|---|---|
| Enzyme elevated | Number elevated out of total | Lactic de-hydro-genase | Oxalo-acetic transa-minase | Aldolase | 6-phospho-gluconate dehydro-genase |
| 6-phospho-gluconate dehydrogenase . | 11 of 12 | 11 of 11 | 9 of 10 | 9 of 11 | — |
| Lactic dehydrogenase . | 11 of 12 | — | 9 of 10 | 9 of 11 | 11 of 11 |
| Oxaloacetic transaminase . | 9 of 11 | 9 of 9 | — | 8 of 9 | 11 of 11 |
| Aldolase . | 10 of 12 | 10 of 10 | 9 of 10 | — | 10 of 10 |

TABLE XI.—*Mixed Enzymes in the Vaginal Fluid of Women with Carcinoma of the Body and with Carcinoma of the Ovary*

| Enzyme units per gram dry weight | | | |
|---|---|---|---|
| 6-phosphogluconate dehydrogenase | Serum glutamic oxaloacetic transaminase | Lactic dehydrogenase | Aldolase |
| | *Carcinoma of the body* | | |
| 216 | 36,000 | 60,000 | 1,959 |
| 19·2 | 800 | 10,000 | 4,030 |
| 31·2 | 7,000 | 30,000 | 320 |
| 763 | — | 48,000 | 5,860 |
| 990 | — | 35,000 | 7,510 |
| | *Carcinoma of the ovary* | | |
| nil | nil | 2,500 | nil |
| 1459 | 1,400 | — | 13,790 |
| 341 | — | 32,500 | 2,310 |
| 158 | — | 8,000 | 1,470 |

In 4 samples from carcinoma of the ovary, no advantage was gained by using more than one enzyme. Three out of four cases were associated with elevated 6-phosphogluconate dehydrogenase, while one case was detected by lactic dehydrogenase, and one by aldolase.

### DISCUSSION

In this series the enzymes have all been compared with 6-phosphogluconate dehydrogenase. The number of enzymes studied depends on the amount of vaginal fluid studied. In general it may be said that women with a gynaecological lesion tend to have a higher secretion of vaginal fluid, thus in this series there will tend to be a greater number of cases with elevated levels unassociated with carcinoma. It is thought that this increased secretion is caused by an inflammatory exudate.

*Results with lactic dehydrogenase*

*Normal women.*—Lactic dehydrogenase gives a slightly higher incidence of abnormal or elevated activities in this group of women than 6-phosphogluconate dehydrogenase, but a very much higher incidence of detectable enzyme than 6-phosphogluconate dehydrogenase. Unlike 6-phosphogluconate dehydrogenase, this enzyme appears to be rarely absent from the vagina. From Fig. 1 it would appear that in normal women if the 6-phosphogluconate dehydrogenase is elevated, then the activity of lactic deydrogenase is related to the amount of 6-phosphogluconate dehydrogenase.

*Benign gynaecological lesions.*—One can have a moderate amount of lactic dehydrogenase in the absence of 6-phosphogluconate dehydrogenase in women with benign gynaecological lesions. If there is an elevated 6-phosphogluconate dehydrogenase, lactic dehydrogenase tends to be higher than in those with low 6-phosphogluconate dehydrogenase levels. In these samples the two enzymes gave a similar number of elevated enzyme levels in the benign cases.

*Gynaecological malignancy.*—Lactic dehydrogenase gave similar results to 6-phosphogluconate dehydrogenase. In these cases there was a closer relationship between the amount of 6-phosphogluconate dehydrogenase and the amount of lactic dehydrogenase detected. It thus appears that little is gained by the use of lactic dehydrogenase instead of 6-phosphogluconate dehydrogenase. In Table V the results for the two enzymes are shown. It was found that 3 out of 4 carcinomas *in situ* would have been detected when both were used ; there would have been no difference in invasive cervical carcinoma but 5 out of 6 carcinomas of the corpus uteri would have been detected. No difference would have been found with ovarian carcinomas. It is possible that the combination of 6-phosphogluconate dehydrogenase with lactic dehydrogenase might be an advantage.

*Results with glutamic oxaloacetic transaminase*

In normal women the oxaloacetic transaminase gave similar results to the 6-phosphogluconate dehydrogenase. In cases with benign gynaecological lesions the oxaloacetic transaminase gave a slightly higher incidence of false positive. In 20 cases of malignancy, one obtained largely similar results. Unfortunately only one case of carcinoma *in situ* was studied.

*Results with aldolase*

Using 2,500 units per gram dry weight, only 1 of 21 normal cases had levels above 2,500, thus 95% of normal cases came below this level. Almost all the women had detectable aldolase values in their vaginal fluids. In this way it would appear to be similar to lactic dehydrogenase. In cases with gynaecological lesions, aldolase gave a lower incidence of elevated levels than 6-phosphogluconate dehydrogenase. It did not seem to be so markedly affected by the trichomonas vaginalis infection or menstruation as 6-phosphogluconate dehydrogenase. In malignancy the results are not as reliable as those with 6-phosphogluconate dehydrogenase.

*Results with glucose-6-phosphate dehydrogenase*

In established carcinoma it was more common for the vaginal fluid glucose-6-phosphate dehydrogenase activity to be elevated to a greater extent than the activity of 6-phosphogluconate dehydrogenase. This finding is in agreement with the work on hepatoma tissue. Unfortunately this finding is not invariable and appears to be of no diagnostic value.

In eight established carcinomas there were only 2 cases in which the glucose-6-phosphate dehydrogenase was not elevated to a greater extent than the 6-phosphogluconate dehydrogenase activity. In both these cases 6-phosphogluconate activities were diagnostic. In a third case both enzymes were not diagnostic. Little seems to be gained by the estimation of both dehydrogenases.

In benign conditions the situation was reversed. In only 3 cases was glucose-6-phosphate dehydrogenase elevated to a greater degree than the 6-phosphogluconate dehydrogenase. The most use the simultaneous study of the two dehydrogenases might have would be to eliminate some of the false positive results.

*Results in multiple enzyme studies*

In normal women it was found that the 6-phosphogluconate dehydrogenase activity correlates with the other enzyme activity. If this enzyme is not raised it is very rare for any of the others to be raised. In cases with gynaecological lesions the enzyme activities parallel each other. The use of the group of enzymes did not enable one to eliminate any false positive results.

It appears that in these carcinomas, the corpus uteri is the only situation in which multiple studies were of diagnostic advantage.

### CONCLUSION

It appears that the use of any of these enzymes by themselves did not improve on 6-phosphogluconate dehydrogenase. The use of a multiple screen by the four enzymes (lactic dehydrogenase, glutamic oxaloacetic transaminase, 6-phosphogluconate dehydrogenase, and aldolase) did not significantly improve on the sole use of 6-phosphogluconate dehydrogenase. It is possible that a combination of 6-phosphogluconate dehydrogenase and lactic dehydrogenase might prove a successful combination.

6-phosphogluconate dehydrogenase has the advantage of not being present in samples of vaginal fluid from normal women. Thus a clear cut distinction can be more easily made. It still has the disadvantage of missing certain cases of carcinoma *in situ.*

### SUMMARY

Multiple enzyme studies have been made of the vaginal fluid in benign and malignant disease of the female genital tract. The results were compared with 6-phosphogluconate dehydrogenase. It appears that none of these enzymes improved on 6-phosphogluconate dehydrogenase.

### REFERENCES

Barrett, H. and Gibson, A. (1964) *Proc. Ass. clin. Biochem.*, **3**, 6.

Bierman, H. R., Hill, B. R., Reinhardt, L., and Emory, E. (1957) Cancer Research, **17**, 660.

Bonham, D. G. and Gibbs, D. F. (1962) *Br. med. J.*, ii, 823.

Bruns, F. (1954) *Biochem. Z.*, **325**, 156.

DYER, H. M., GALLINO, P. M., ENSFIELD, B. J. AND MORRIS, H. P. (1961) *Cancer Res.*, **21**, 1522.
FLEISHER, G. A. (1959) *Gastroenterology*, **37**, 325.
GLOCK, G. E. AND MCLEAN, P. (1953) *Biochem. J.*, **55**, 404.
HILL, B. R. AND LEVI, C. (1954) *Cancer Res.* **14**, 513.
HORROCKS, J. E., KING, J., WAIND, A. P. B. AND WARD, I. J. (1962) *J. clin. Path.*, **15**, 57.
KARMEN, A., WROBLEWSKI, F. AND LADUE, J. S. (1955) *J. clin. Invest.*, **34**, 126.
LATNER, A. L. (1964) *Proc. Ass. clin. Biochem.*, **3**, 120.
MUIR, G. G. AND CANTI, G. (1966) *J. Obstet. Gynaec. Br. Commonw.*, In press.
ODELL, L. D. AND BURT, J. C. (1949) *Cancer Res.* **9**, 362.
SIBLEY, J. A. AND LEHNINGER, A. L. (1949) *J. natn. Cancer Inst.*, **9**, 303.
WARBURG, O. AND CHRISTIAN, W. (1943) *Biochem, Z.*, **314**, 399.
WHITE, L. J. (1958) *J. natn. Cancer Inst.*, **21**, 671.
WROBLEWSKI, F. AND LADUE, J. S. (1955) *Proc. Soc. exp. Biol. Med.*, **90**, 210.
WROBLEWSKI, F. AND WROBLEWSKI, R. (1958) *Ann. intern Med.*, **98**, 113.


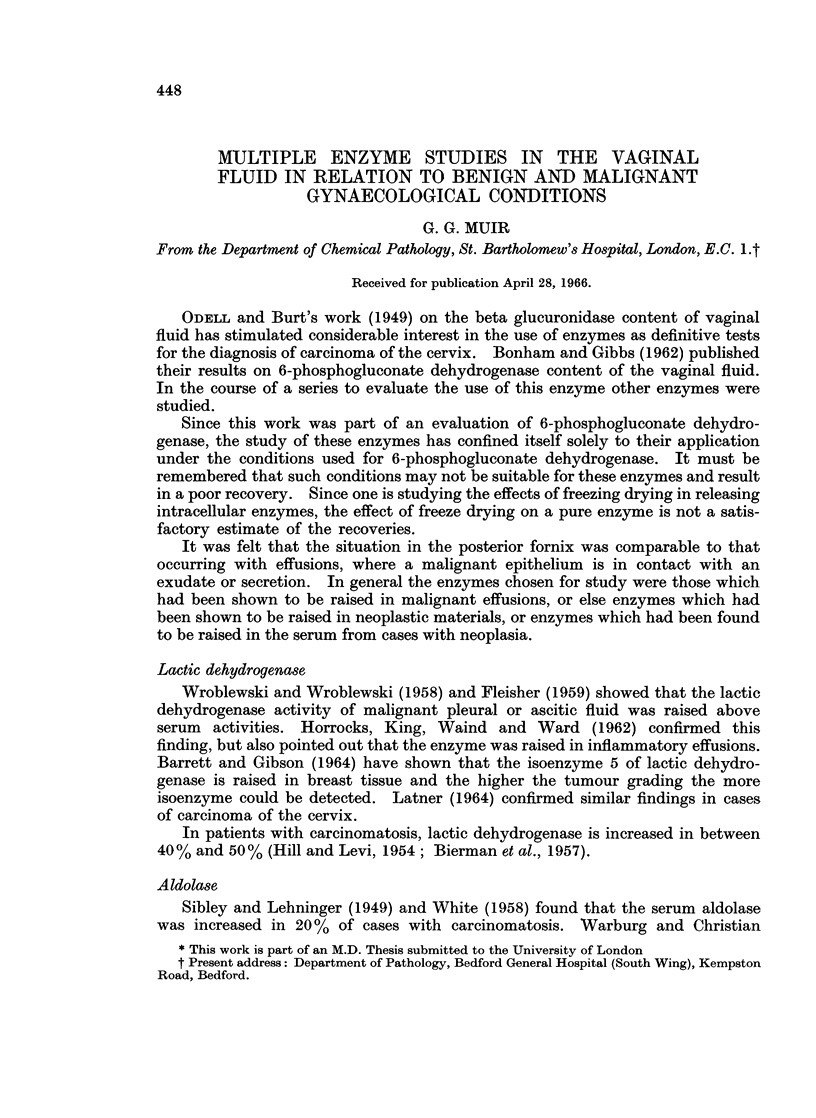

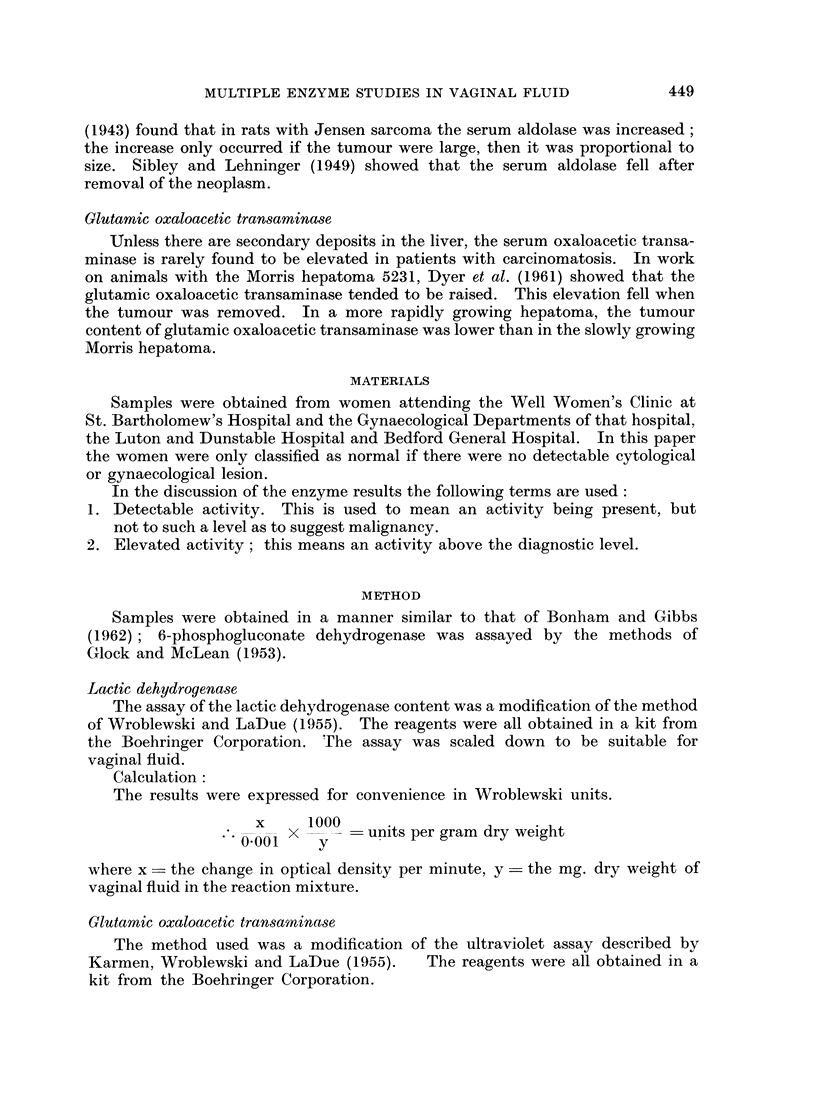

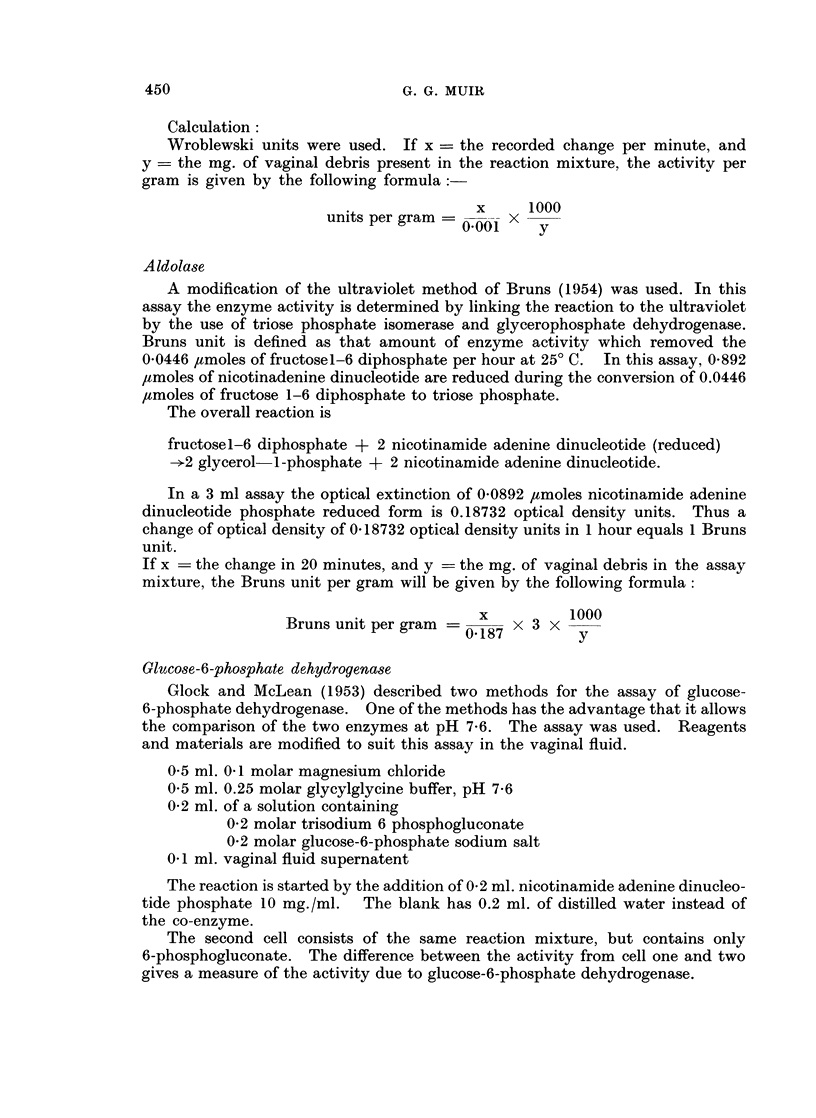

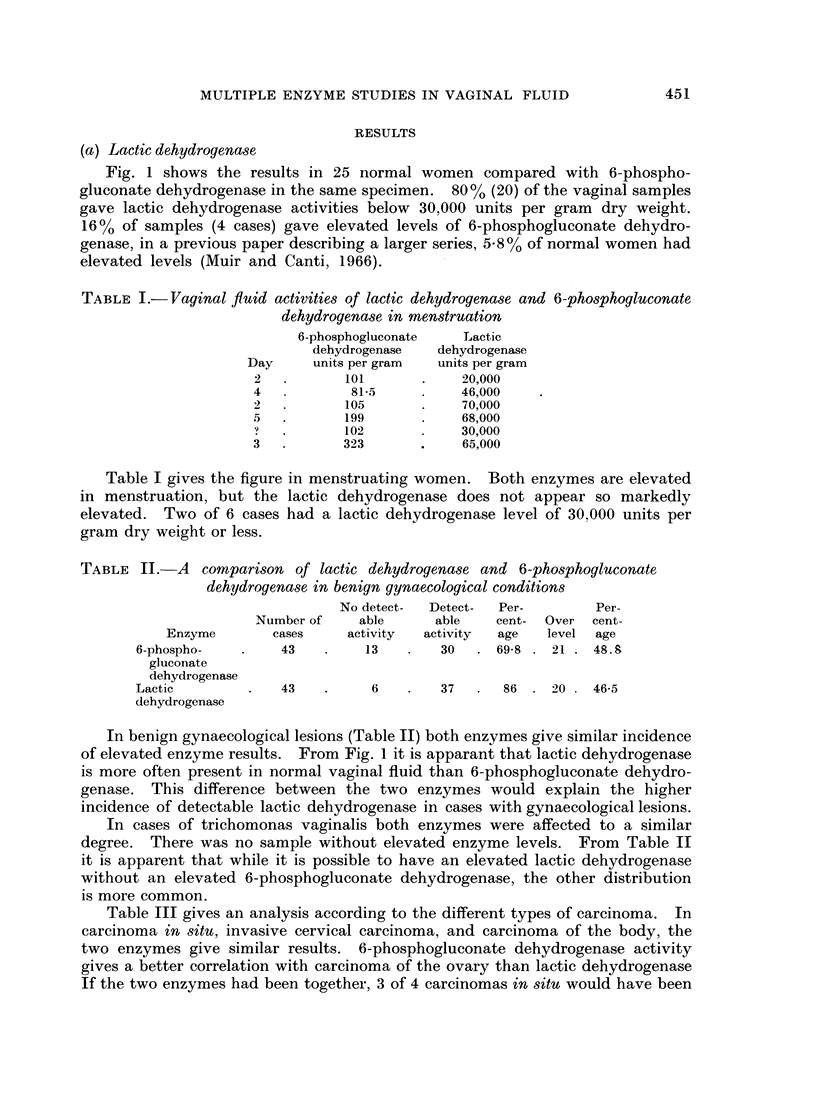

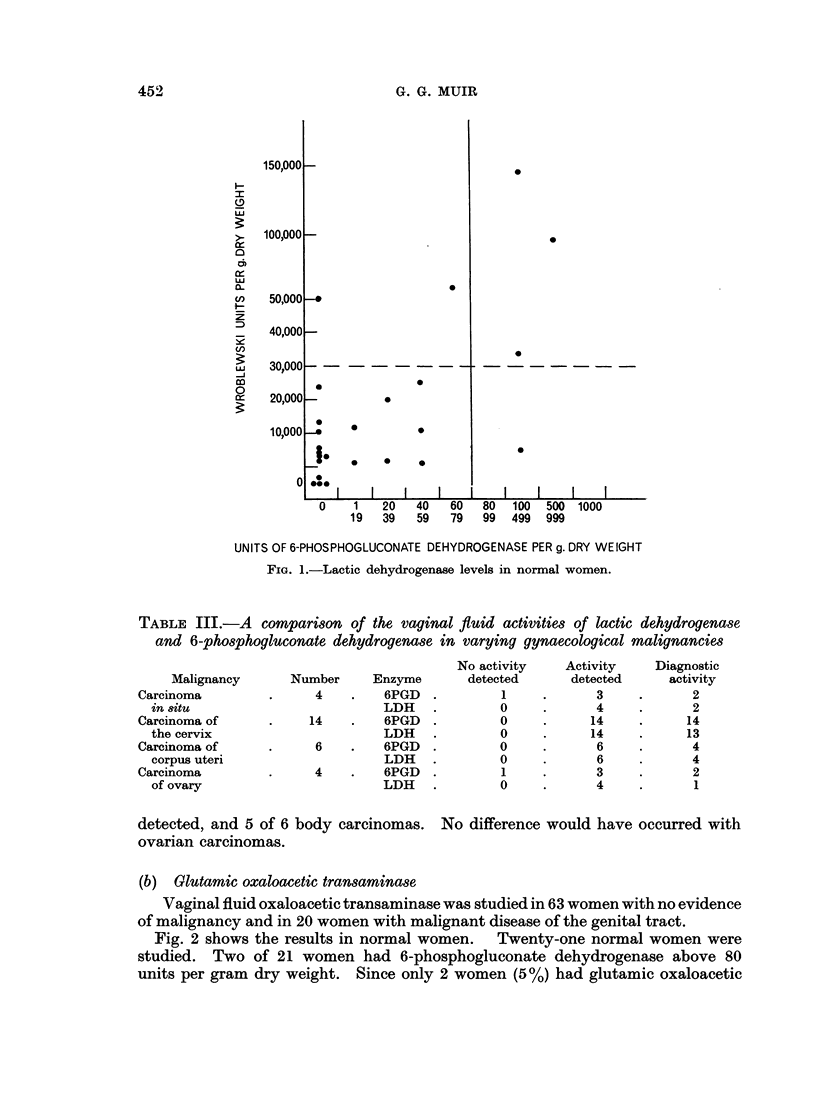

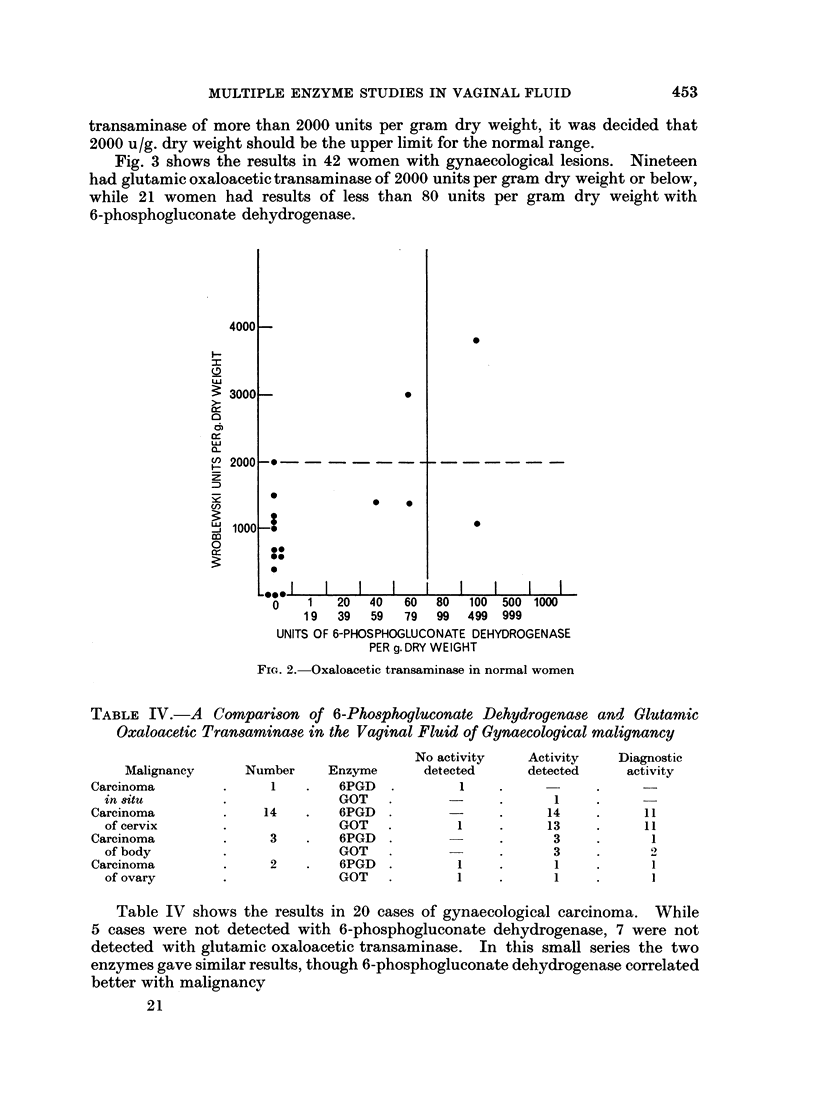

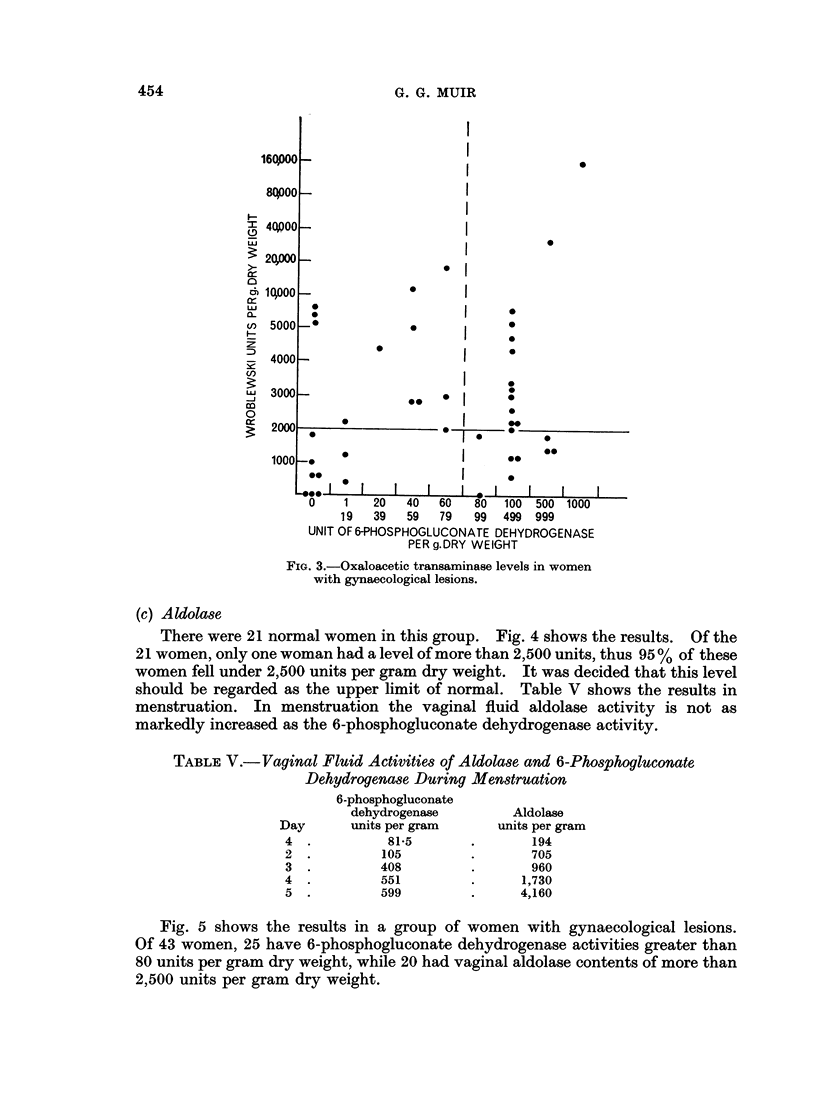

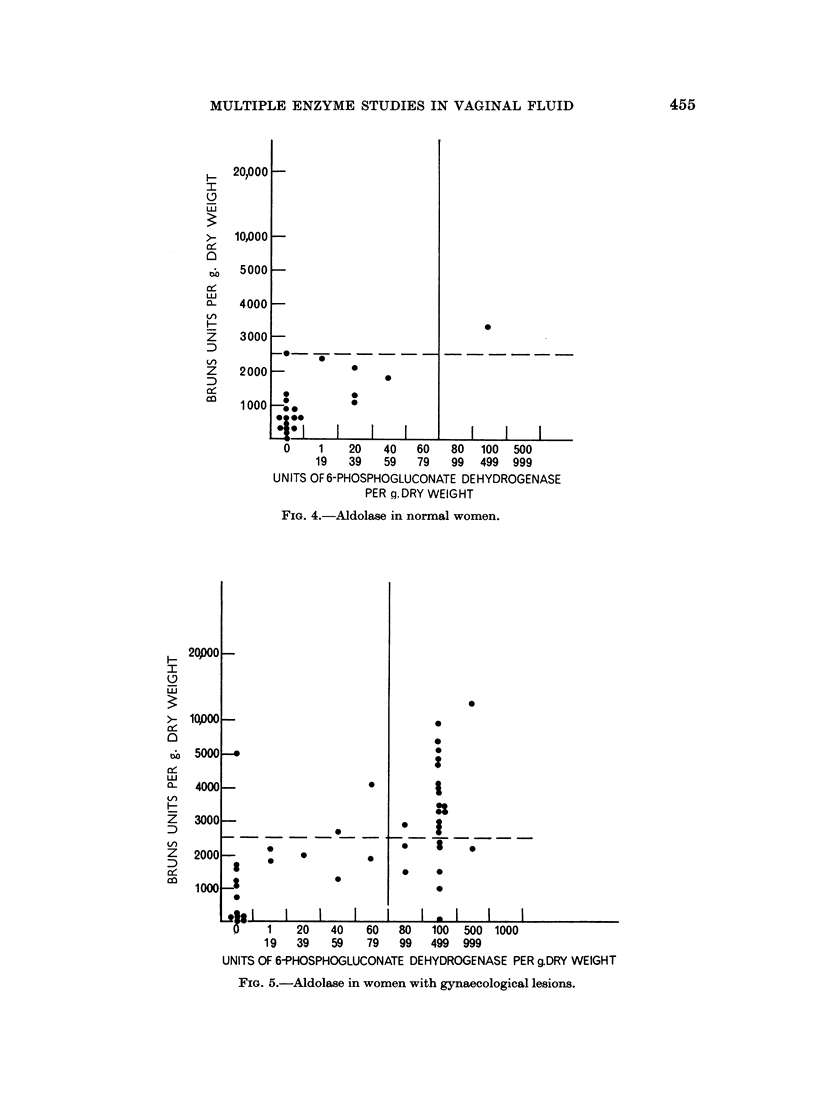

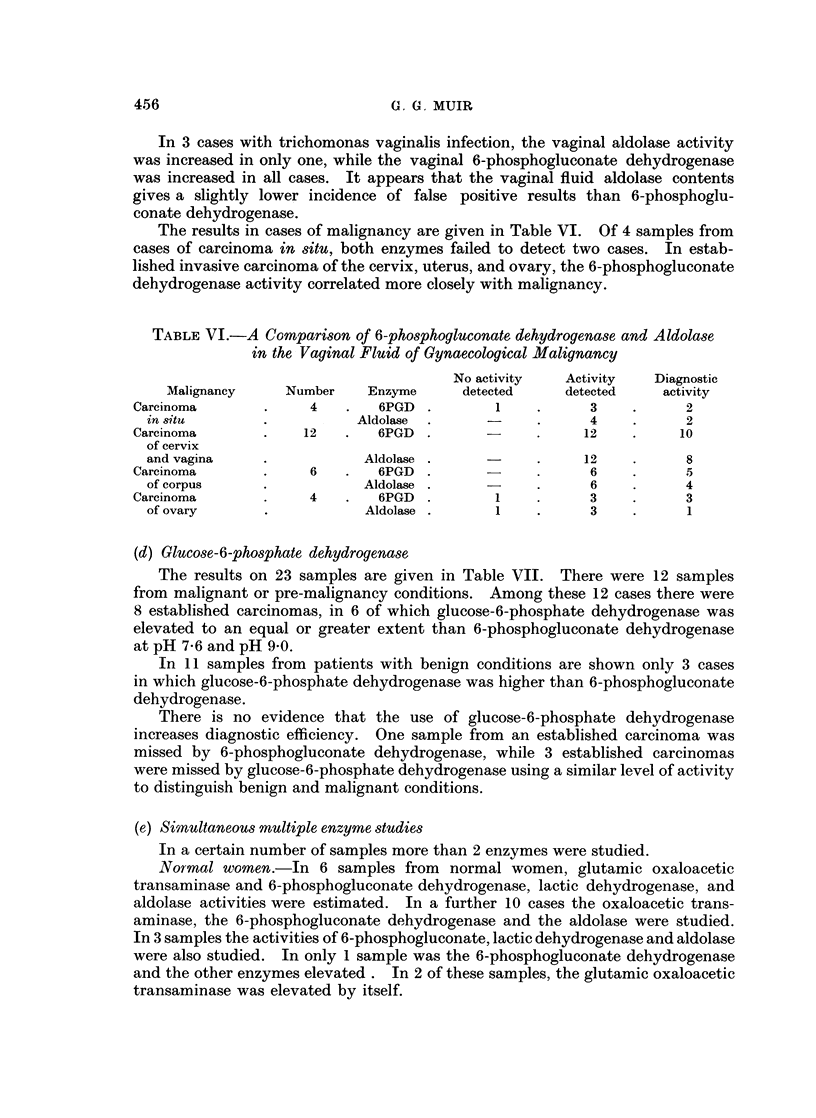

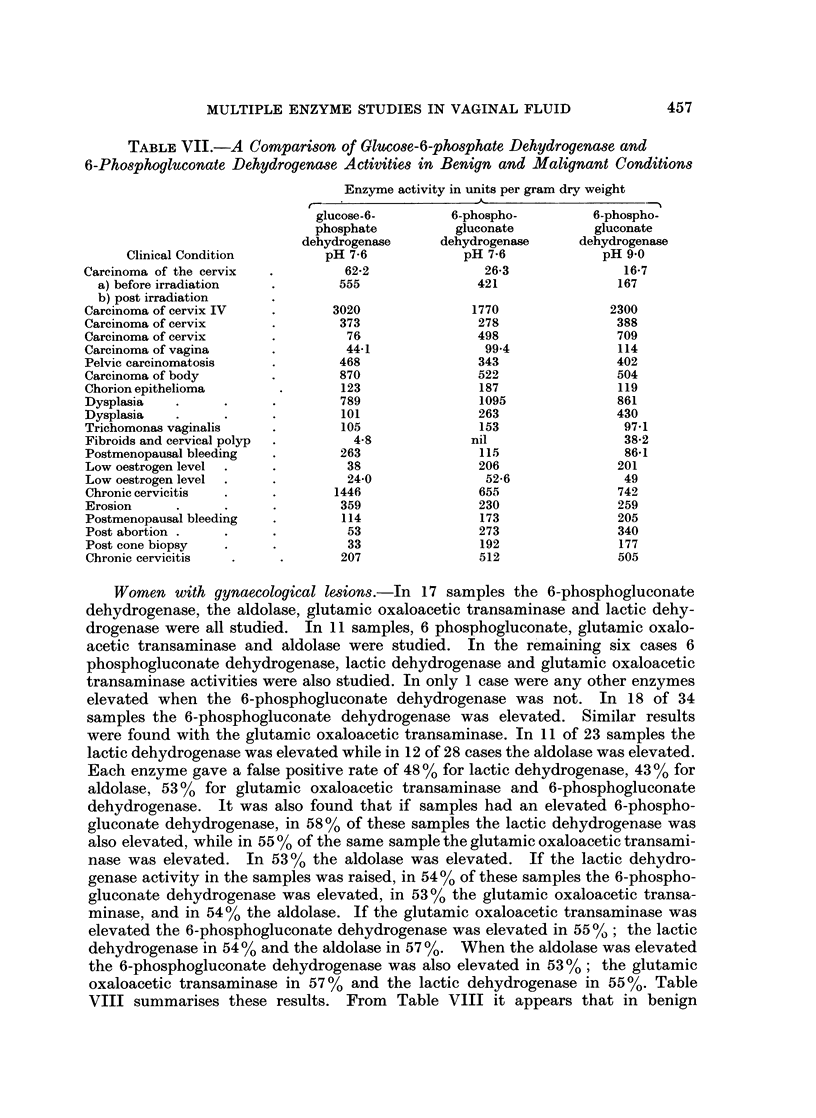

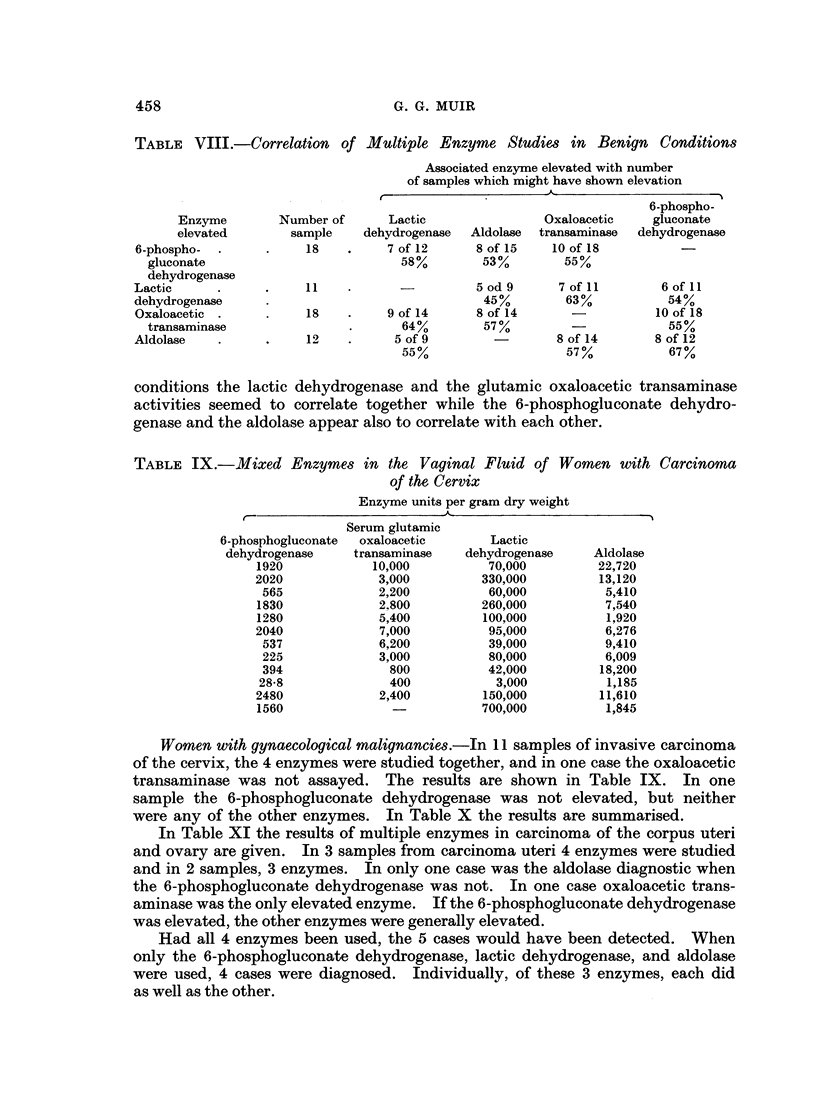

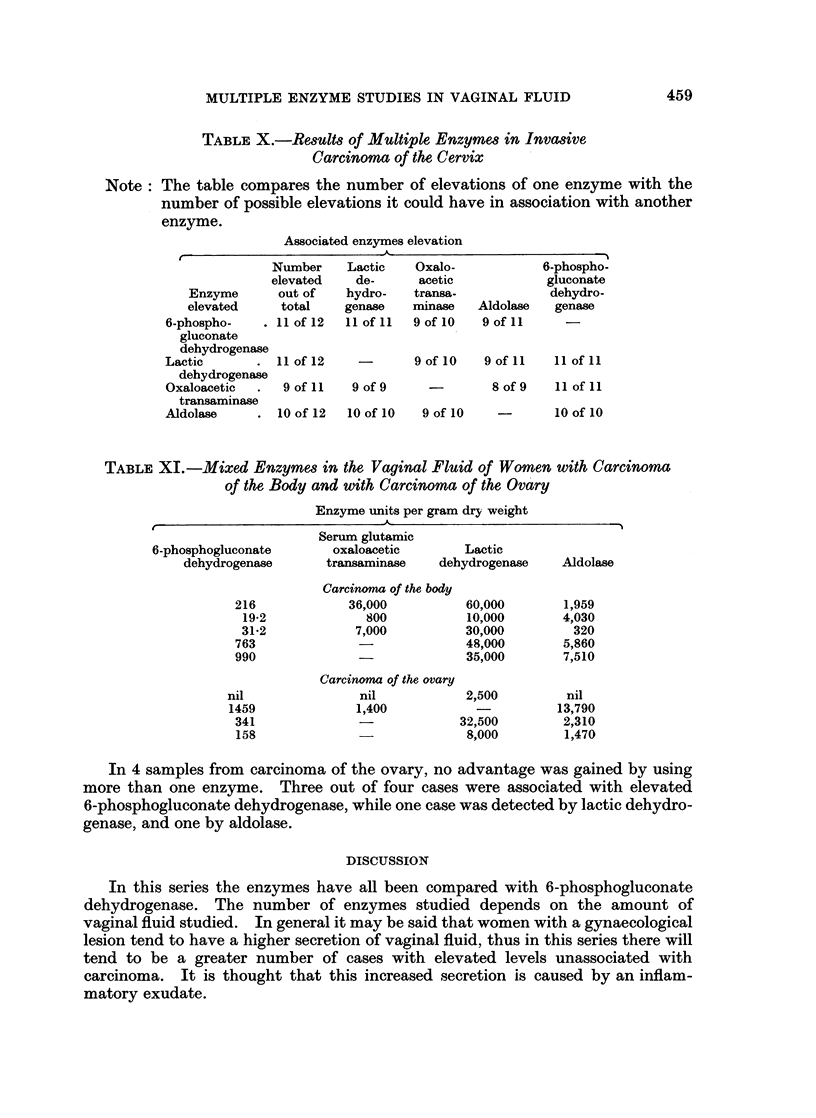

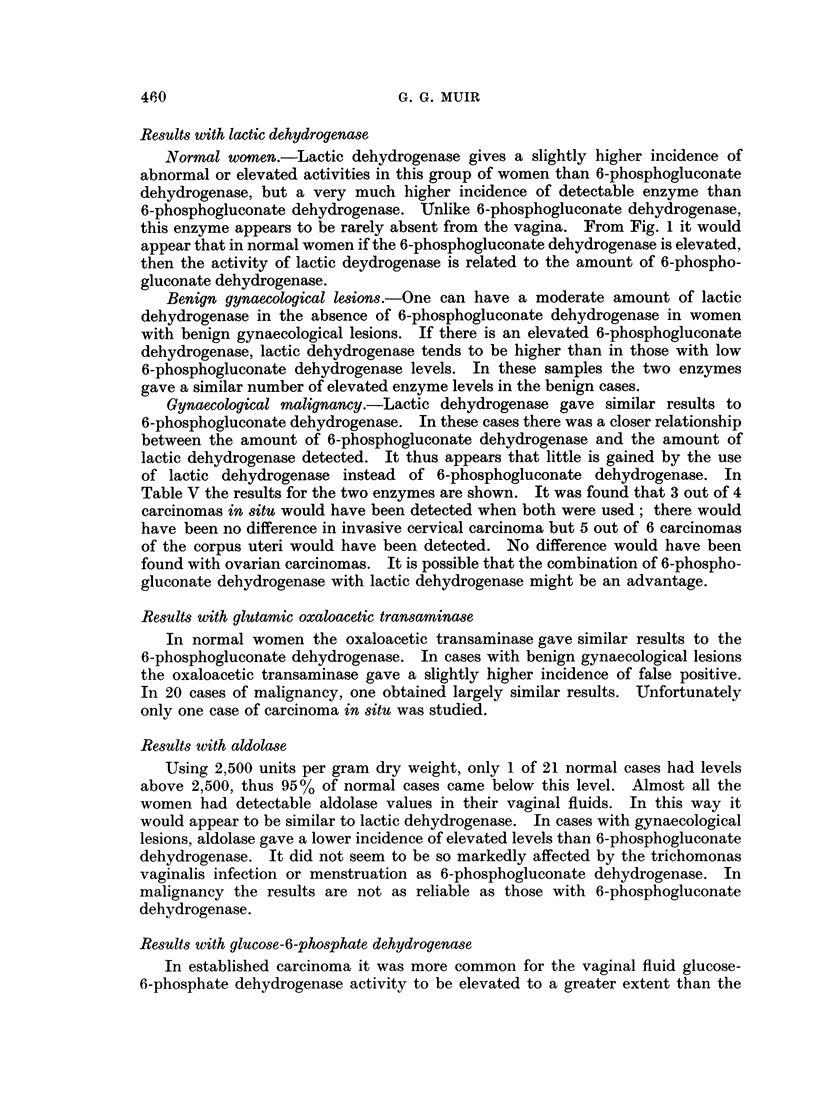

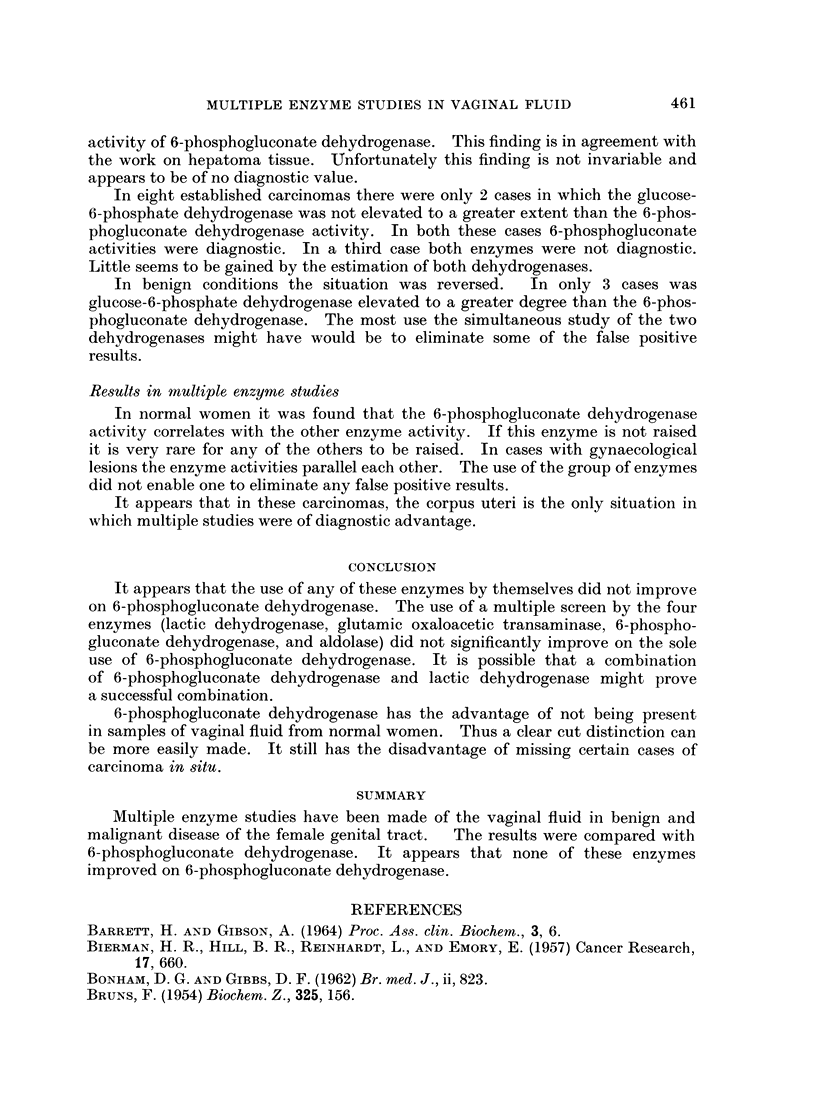

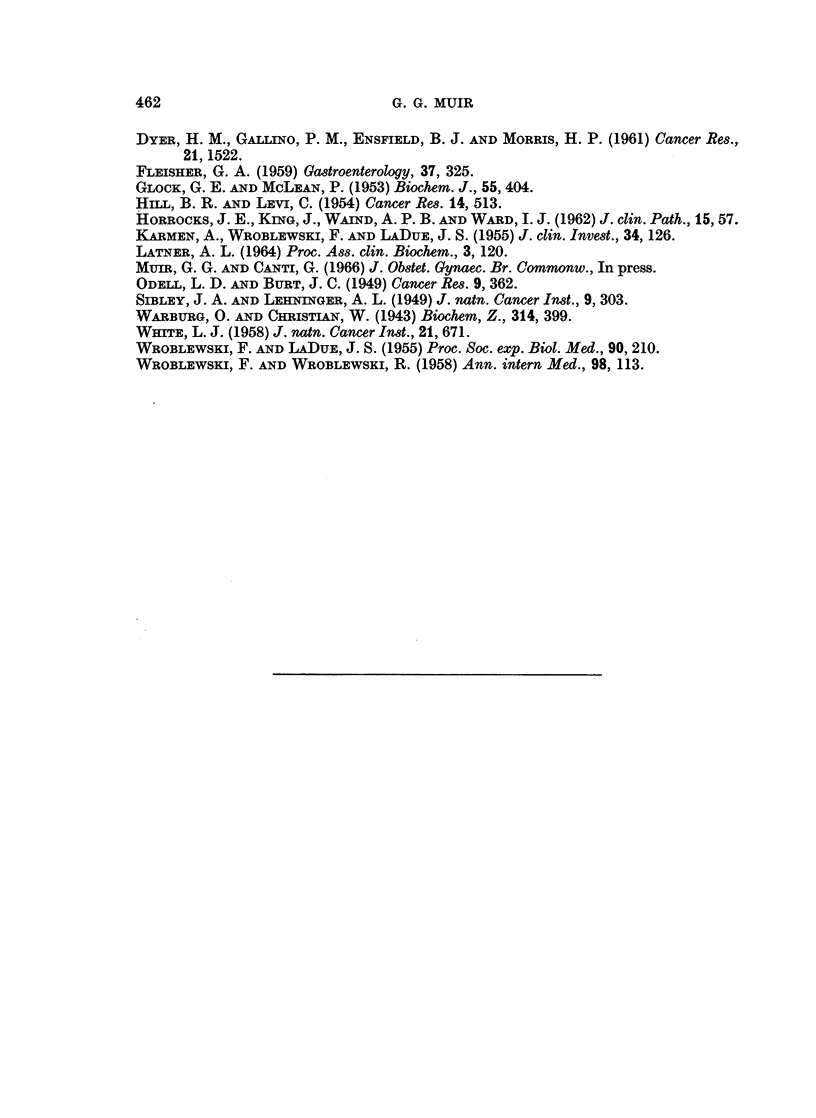


## References

[OCR_01335] BIERMAN H. R., HILL B. R., REINHARDT L., EMORY E. (1957). Correlation of serum lactic dehydrogenase activity with the clinical status of patients with cancer, lymphomas, and the leukemias.. Cancer Res.

[OCR_01339] BONHAM D. G., GIBBS D. F. (1962). A new enzyme test for gynaecological cancer. 6-Phosphogluconate dehydrogenase activity in vaginal fluid.. Br Med J.

[OCR_01340] BRUNS F. (1954). Bestimmung und Eigenschaften der Serumaldolase.. Biochem Z.

[OCR_01344] DYER H. M., GULLINO P. M., ENSFIELD B. J., MORRIS H. P. (1961). Transaminase activities of liver tumors and serum.. Cancer Res.

[OCR_01348] FLEISHER G. A., BARTHOLOMEW L. G., CAIN J. C., ROVELSTAD R. A. (1959). Ascites. II. The value of determinations of enzymes in the study of ascitic fluid.. Gastroenterology.

[OCR_01351] HILL B. R., LEVI C. (1954). Elevation of a serum component in neoplastic disease.. Cancer Res.

[OCR_01353] HORROCKS J. E., KING J., WAIND A. P., WARD J. (1962). Lactate dehydrogenase activity in the diagnosis of malignant effusions.. J Clin Pathol.

[OCR_01354] KARMEN A., WROBLEWSKI F., LADUE J. S. (1955). Transaminase activity in human blood.. J Clin Invest.

[OCR_01364] WROBLEWSKI F., LADUE J. S. (1955). Lactic dehydrogenase activity in blood.. Proc Soc Exp Biol Med.

